# Dosimetric Study of Deep Learning-Guided ITV Prediction in Cone-beam CT for Lung Stereotactic Body Radiotherapy

**DOI:** 10.3389/fpubh.2022.860135

**Published:** 2022-03-22

**Authors:** Shujun Zhang, Bo Lv, Xiangpeng Zheng, Ya Li, Weiqiang Ge, Libo Zhang, Fan Mo, Jianjian Qiu

**Affiliations:** Department of Radiation Oncology, Huadong Hospital, Fudan University, Shanghai, China

**Keywords:** 4DCT, CBCT (cone beam computed tomography), SBRT (stereotactic body radiation therapy), deep learning, Mask R-CNN

## Abstract

**Purpose:**

The purpose of this study was to evaluate the accuracy of a lung stereotactic body radiotherapy (SBRT) treatment plan with the target of a newly predicted internal target volume (ITV_predict_) and the feasibility of its clinical application. ITV_predict_ was automatically generated by our in-house deep learning model according to the cone-beam CT (CBCT) image database.

**Method:**

A retrospective study of 45 patients who underwent SBRT was involved, and Mask R-CNN based algorithm model helped to predict the internal target volume (ITV) using the CBCT image database. The geometric accuracy of ITV_predict_ was verified by the Dice Similarity Coefficient (DSC), 3D Motion Range (R_3D_), Relative Volume Index (RVI), and Hausdorff Distance (HD). The PTV_predict_ was generated by ITV_predict_, which was registered and then projected on free-breath CT (FBCT) images. The PTV_FBCT_ was margined from the GTV on FBCT images gross tumor volume on free-breath CT (GTV_FBCT_). Treatment plans with the target of Predict planning target volume on CBCT images (PTV_predict_) and planning target volume on free-breath CT (PTV_FBCT_) were respectively re-established, and the dosimetric parameters included the ratio of the volume of patients receiving at least the prescribed dose to the volume of PTV (R_100%_), the ratio of the volume of patients receiving at least 50% of the prescribed dose to the volume of PTV in the Radiation Therapy Oncology Group (RTOG) 0813 Trial (R_50%_), Gradient Index (GI), and the maximum dose 2 cm from the PTV (D_2cm_), which were evaluated *via* Plan_4DCT_, plan which based on PTV_predict_ (Plan_predict_), and plan which based on PTV_FBCT_ (Plan_FBCT_).

**Result:**

The geometric results showed that there existed a good correlation between ITV_predict_ and ITV on the 4-dimensional CT [ITV_4DCT_; DSC= 0.83 ±0.18]. However, the average volume of ITV_predict_ was 10% less than that of ITV_4DCT_ (*p* = 0.333). No significant difference in dose coverage was found in V_100%_ for the ITV with 99.98 ± 0.04% in the ITV_4DCT_ vs. 97.56 ± 4.71% in the ITV_predict_ (*p* = 0.162). Dosimetry parameters of PTV, including R_100%_, R_50%_, GI and D_2cm_ showed no statistically significant difference between each plan (*p* > 0.05).

**Conclusion:**

Dosimetric parameters of Plan_predict_ are clinically comparable to those of the original Plan_4DCT._ This study confirmed that the treatment plan based on ITV_predict_ produced by our model could automatically meet clinical requirements. Thus, for patients undergoing lung SBRT, the model has great potential for using CBCT images for ITV contouring which can be used in treatment planning.

## Introduction

For patients with early-stage lung cancer, stereotactic body radiotherapy (SBRT) has become one of the primary treatment options. It has been proven to significantly improve the tumor control and overall survival rate of patients with early-stage lung cancer (1–4).

Currently, the most popular treatment method is to use four-dimensional CT (4DCT) imaging to generate the internal target volume (ITV) contour, which expresses the volume of a tumor moving throughout a patient's breathing. This ITV contour from a four-dimensional averaged (4DAVG) image is used to generate a radiation treatment plan (5).

With the 4DCT technique, image acquisition is associated with the patient's breathing curve. The limitations of 4DCT are as follows: (1) Required high patient compliance as an irregular breathing curve can reduce the image quality and affect the accuracy of tumor contouring (6); (2) Complicated and professional operation as it requires a longer time to acquire 4DCT images, which could increase the instability and randomness of the simulation (7); (3) Low popularity as it is estimated that less than half of radiotherapy centers are equipped with four-dimensional scanners (8). Overall, these limits may potentially reduce the SBRT accuracy in treatment.

Conversely, CBCT has high popularity and is conventionally equipped in a linear accelerator (9). In addition, it is mainly used to compare the anatomical landmarks from treatment planning CT images in clinical practice, which are used to determine intra/inter-fraction motion (10). CBCT rotates 360° around the patient's body and then finishes the CT image scanning within a period of time (~1 min) which includes 10–12 respiration time phases and the motion trajectory of the tumor. The limitations of CBCT are as follows. First, poor image quality is the main factor affecting radiation oncologist determination of lung tumor volume (11). Second, due to a prominent amount of and artifacts, the dose calculation based on CBCT images may be inaccurate for treatment planning (12).

In recent years, “deep learning” has been extensively used in medical image processing. Among them, convolutional neural networks (CNNs) are the primary methods of target detection and segmentation (13–16). Mask R-CNN is a simple, flexible, commonly used framework for object instance segmentation, and is popular in medical image processing (17). Bouget et al. used the detection of mediastinal lymph nodes in CT images for lung cancer staging while enabling good instance detection (18). Zhang et al. successfully used Mask R-CNN to detect lung tumors on PET images, which has more effectively and precisely while suitably avoiding incorrect detection of tumors (19). Some previous studies used Mask R-CNN on segmentation, such as detection and classification the breast tumors on sonograms (20) and brain tumor segmentation for dynamic susceptibility contrast-enhanced perfusion imaging (21). These studies also show the great potential of Mask R-CNN in object detection and segmentation, presenting a possibility of it being used in clinical applications of medical images in the future.

Our preliminary research confirmed that the upgraded Mask R-CNN model could predict the ITV with CBCT image accuracy (22). Meanwhile, SBRT delivers high radiation doses to the tumor target in a hypo-fractionated area with a minimum dose to the tissue around the target area (19). Therefore, dosmetric research for lung SBRT is important. This study aimed to evaluate the dose accuracy of a lung SBRT treatment plan with ITV_predict_ and the feasibility of its clinical application.

## Materials and Methods

### Patient Data

Forty-five lung cancer patients (average age was 68 years, range: 55–86 years) who underwent SBRT at Huadong Hospital from January 2020 to July 2021 were randomly selected for this study.

### Image Acquisition

Each patient's free-breath CT (FBCT) was scanned by a Siemens Somatom Definition AS® CT scanner (Siemens Somatom Sensation, Munich, Germany) with a pitch of 1.5 and slice thickness of 1 mm. 4DCT images were acquired with the additional assistance of Varian real-time position management (RPM) (Varian Medical Systems, Palo Alto, USA) using the same scanning parameters. The first treatment fraction of CBCT images were acquired in our CBCT system (100 kVp and 100 mAs, rotated at 360° with a speed of 6° per second), equipped with a VarianVitalBeam^TM^ linear accelerator (Varian Medical Systems, Palo Alto, USA).

### ITV Acquisition

**Figure 1:**
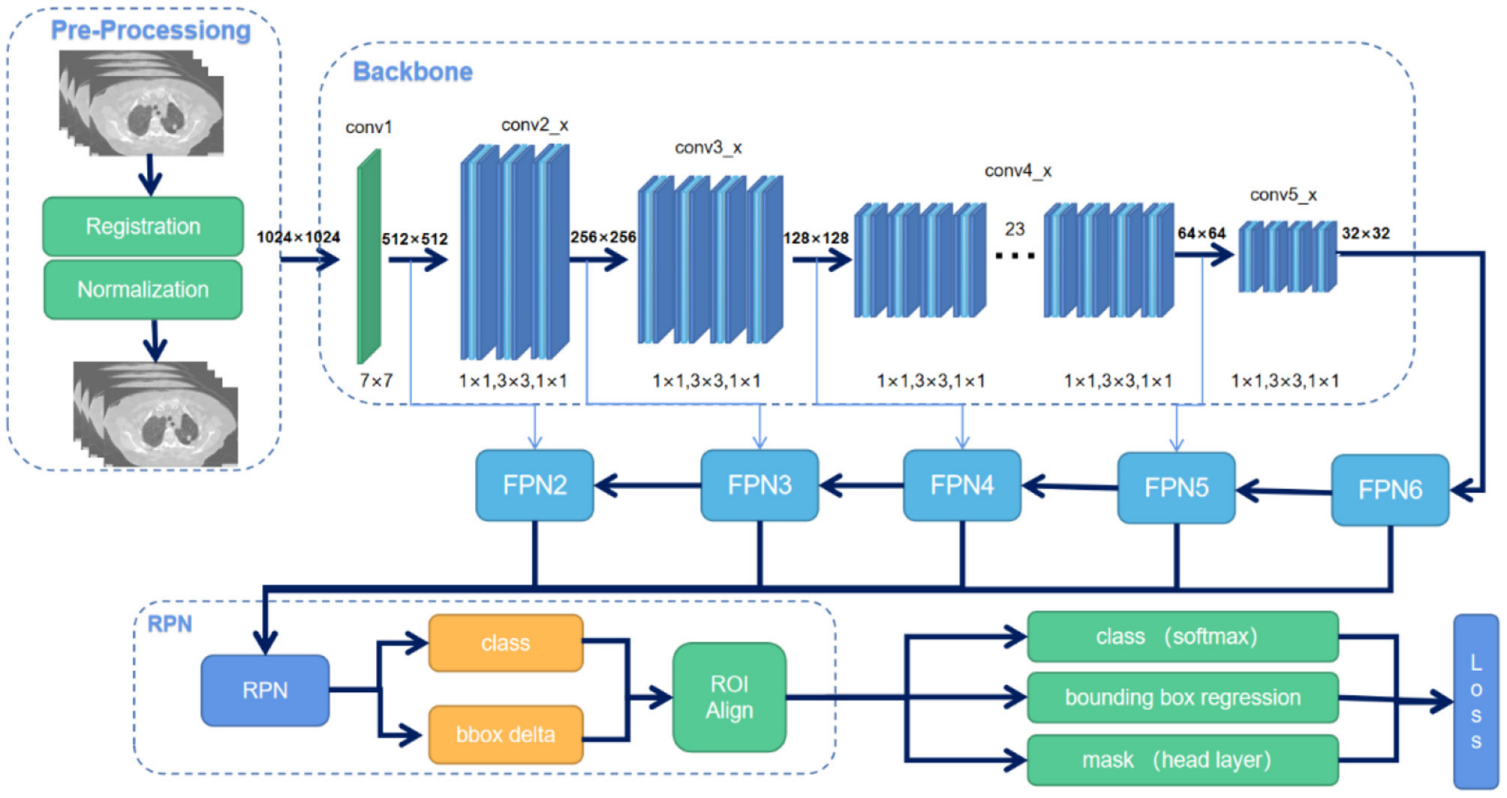
Overview of the Mask R-CNN workflow^22^. There are two steps. First, it generates proposals for lung tumor regions based on the input cone-beam CT (CBCT) image. Second, it generates a tumor pixel-level mask based on the proposal created in the first step.

The gross tumor volume (GTV) on FBCT images (GTV_FBCT_) and the ITV on 4DAVG (ITV_4DCT_) images were contoured by two radiation oncologists with expertise in lung tumors. Our model, using the Mask R-CNN algorithm with a convolutional block attention module (CBAM) module embedded, was used to automatically establish a newly predicted ITV (ITV_predict_) using the CBCT image database (18). Then, we registered the CBCT image and FBCT image and projected the ITV_predict_ on the FBCT image to calculate the dose. The model workflow is shown in Figure 1.

### Treatment Plan

PTV_4DCT_ and PTV_predict_ were defined as ITV margins of 5 mm on 4DAVG images and FBCT images, respectively. PTV_FBCT_ was defined as GTV_FBCT_ with a margin of 10 mm in the craniocaudal (CC) direction and 5 mm in the left-right (LR) and anterior-posterior (AP) directions in the FBCT image.

**Figure 2:**
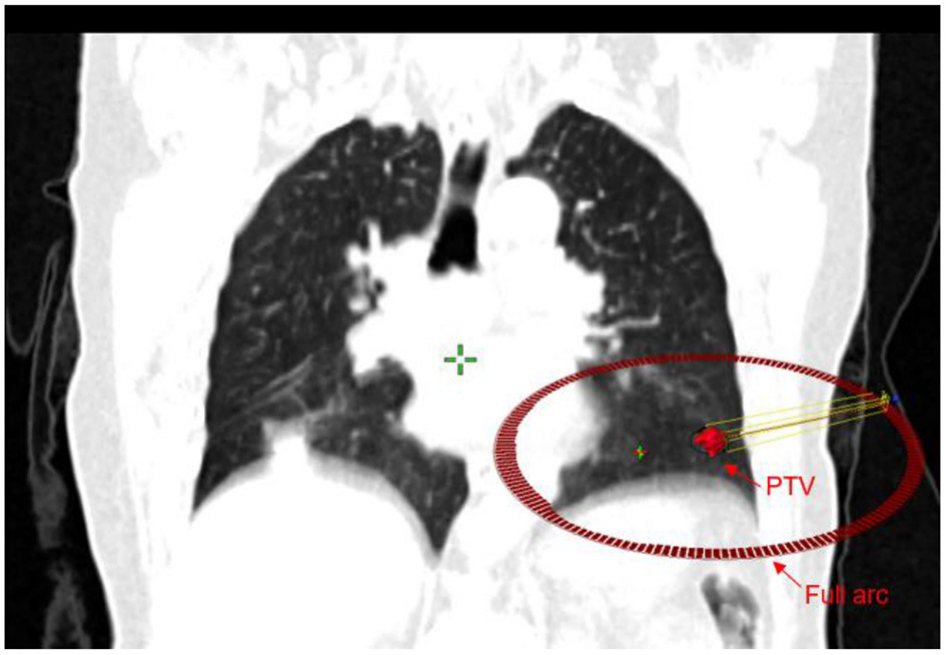
Volumetric-modulated arc therapy (VMAT) plans with a full arc.

All patient plans were replanned in the Varian Eclipse® system (version 15.5), which was generated by a full arc and was used depending on the location and anatomic relationships of the tumors and normal tissues (Figure 2), by our experienced medical physicists. We used a 6 MV-FFF (DR: 1400 MU/min) energy and the anisotropic analytical dose calculation algorithm (AAA) with a 2.5 mm^3^ calculating grid in all plans. All patients received prescription of 60 grays (Gy) in 10 fractions (6 Gy per fraction) for over 2 weeks. The planning objectives aimed to cover the PTV with 95% of the prescribed dose in all plans.

### Geometry Evaluation Parameters for the Prediction Model

The Dice Similarity Coefficient (DSC), 3D Motion Range (R_3D_), Relative Volume Index (RVI), and Hausdorff Distance (HD) were calculated to assess the agreement between ITV_predict_ and ITV_4DCT_. All statistical tests were performed using SciPy (23) in Python.

### PTV Evaluation Parameters

The volume of PTV (V_PTV_, cm^3^), mean dose (D_mean_), the maximum dose received by 2% (D_2%_), and the minimum dose received by 98% of the evaluated PTV volume (D_98%_) were determined. The percent of the PTV receiving 100% of the prescription dose (V_100%_) and the dose covering 95% of PTV (D_95%_) were also calculated.

A steep dose gradient at the margin of the target volume is an important part of the SBRT plan to protect the normal organization. Some parameters for quantification have been reported in the literature, including R_100%_, R_50%_, the Gradient Index (GI), and the maximum dose 2 cm from the PTV (D_2cm_).

### R_100%_

R_100%_ is the ratio of the volume of patients receiving at least the prescribed dose to the volume of PTV (9). When the value of R_100%_ is closer to 1, it means that the dose distribution has more conformity for PTV.


$$R_{100\%}=\frac{V_{100\%}}{V_{PTV}}$$

### R_50%_

R_50%_ was defined as the ratio of the volume of patients receiving at least 50% of the prescribed dose to the volume of PTV in the Radiation Therapy Oncology Group (RTOG) 0813 Trial (9). The R_50%_ is an evaluation index for damage to irradiated normal tissues (24).


$$\begin{array}{l} R_{50\%}=\frac{V_{50\%}}{V_{PTV}} \end{array}$$

### Gradient Index (GI)

The Gradient Index (GI) is defined as the ratio of the volume of the patient receiving at least 50% of the prescription dose to the volume of the patient receiving at least 100% of the prescription dose (25). It was used to measure dose fall-off outside of the PTV. The dose falling off outside the target volume is very important in SBRT, especially as a predictor of complications (26).


$$\begin{array}{l} Gradient\;Indax\;\left(GI\right)=\frac{V_{50\%}}{V_{100\%}} \end{array}$$

### OARs Evaluation Parameters and Treatment Efficiency Parameters

The dosimetric parameter acceptance of normal tissues (27), which refers to the RTOG 0813 Trial, is listed in Table 1. The ITV acquisition time was manually recorded for delineation efficiency and automatically recorded for model, and machine monitor units (Mus) were recorded for treatment efficiency.

**Table 1:** Planning objectives for critical structures.

**Objectives**	**Parameters**	**Limit**
Normal Lung	V 20 Gy	<10%
	V12.5 Gy	<15%
Heart	D_max_	<32.5Gy
Trachea	D_max_	<32.0Gy
Esophagus	D_max_	<35.0Gy
Spinal Cord	D_max_	<25.0Gy

### Statistical Analysis

The statistical significance of the difference between the groups was assessed using 1-way analysis of variance (ANOVA) by SPSS software release 20.0, and the statistical significance was *p* < 0.05.

## Results

### Clinical Characteristics

Patient and tumor characteristics of the 45 patients are described in Table 2.

**Table 2:** Patient and tumor characteristics.

**Parameter**	**Total**
Patients (*n* = 45)	Female = 17, Male = 28
Median age in years (range)	68 (55–86)
Median ITV in cm^3^ (range)	21.42 (0.7–65.7)
Tumor location (*n* = 45)	5 LUL, 13 LLL, 6 RUL, 7 RLL, 14 RML

*LUL, left upper lobe; RUL, right upper lobe; LLL, left lower lobe; RLL, right lower lobe; RML, right middle lobe*.

### Geometry Evaluation

**Figure 3:**
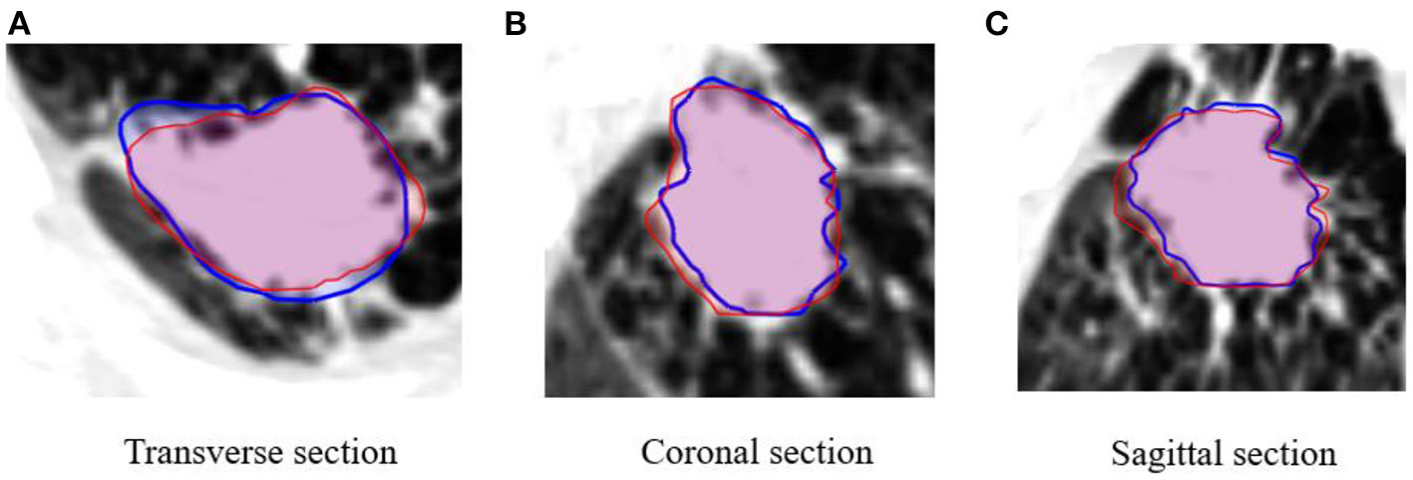
Comparison of contours between ITV on the 4-dimensional CT (ITV_4DCT_, red) and ITV prediction (ITV_predict_, blue) in different views of patient images.

The DSC value between ITV_4DCT_ and ITV_predict_ was 0.83 ± 0.18. The DSC value indicates that ITV_4DCT_ and ITV_predict_ have a good correlation (Table 3). In addition, Figure 3 shows a visual evaluation from different perspectives. ITV_predict_ can outline the patient's tumor contour and is similar to the radiation oncologist contouring (ITV_4DCT_). The visual assessment shows that the results are reasonable.

**Table 3:** Geometry parameters of the patients.

**Parameters**	**DSC**	**R_**3D**_**	**RVI**	**HD**
	**(mean ±SD)**	**(mean ±SD)**	**(mean ±SD)**	**(mean ±SD)**
	0.83 ± 0.18	3.08 ± 2.81	1.14 ± 0.21	19.77 ± 21.59

### ITV Evaluation

The average volumes of the ITV_4DCT_ and ITV_predict_ were 21.41 ± 9.38 cm^3^ and 19.31 ± 8.83 cm^3^, respectively. Compared to ITV_4DCT_, ITV_predict_ reduced ITV volume by 10% on average (*p* = 0.333). No significant difference was found in ITV volume. However, no significant difference was found in V_100%_ for the ITV with 99.98 ± 0.04% in ITV_4DCT_ vs. 97.56 ± 4.71% in ITV_predict_ (*p* = 0.162) (Table 4).

**Table 4:** Calculated prescription dose coverage (V_100_) and dose to 95% (D_95_) of ITV.

**Variables**	**ITV_**4DCT**_**	**ITV_**predict**_**	***P* value**
	**(mean ±SD)**	**(mean ±SD)**	
Volume (cm^3^)	21.41 ± 9.38	19.31 ± 8.83	0.333
V_100%_ (%)	99.98 ± 0.04	97.56 ± 4.71	0.162
D_95%_ (Gy)	63.84 ± 1.55	61.02 ± 5.56	0.207

* A significant difference existed (p < 0.05).

*SD, standard deviation*.

### PTV Evaluation

The PTV evaluation parameters are shown in Table 5. The GI value of Plan_predict_ (GI = 3.98 ± 0.42) was slightly lower than that of Plan_FBCT_ (GI = 4.74 ± 1.01), indicating that the descending gradient of PTV was better than that of Plan_FBCT_ and second to that of Plan_4DCT_ (GI = 3.31 ± 0.89). The R_100%_ value for Plan_4DCT_ (R_100%_ =1.05 ± 0.11) was always lower than that for Plan_predict_ (R_100%_ = 1.08 ± 0.05) and Plan_FBCT_ (R_100%_ = 1.12 ± 0.06), and the results show that Plan_4DCT_ has the best performance and high conformability. However, there was no statistically significant difference between the plans (F = 0.141).

**Table 5:** Dosimetric parameter comparison among Plan_4DCT_, Plan_predict_, and Plan_FBCT_.

**Variables**	**Plan_**4DCT**_ (mean ±SD)**	**Plan_predict_** **(mean ±SD)**	**Plan_**FBCT**_ (mean ±SD)**	***F***	***P***
***PTV***					
Volume (cm^3^)	48.81 ± 38.99	44.84 ± 31.93	43.89 ± 34.05	0.051	0.952
D_98_ (Gy)	45.54 ± 10.72	51.01 ± 6.84	50.88 ± 4.33	0.384	0.685
D_2_ (Gy)	77.21 ± 7.03	78.4 ± 14.89	81.93 ± 12.37	1.430	0.259
V_100_ (%)	94.40 ± 1.80	94.9 ± 0.28	94.9 ± 0.25	0.931	0.408
D_95_ (Gy)	59.76 ± 5.76	59.98 ± 3.62	59.59 ± 7.82	0.662	0.525
R_100%_	1.05 ± 0.11	1.08 ± 0.05	1.12 ± 0.06	0.141	0.872
R_50%_	3.48 ± 0.82	5.25 ± 2.01	4.45 ± 0.70	0.560	0.598
GI	3.31 ± 0.89	3.98 ± 0.42	4.74 ± 1.01	0.573	0.592
D_2cm_ (Gy)	30.27 ± 4.39	31.18 ± 3.46	33.46 ± 2.24	0.070	0.933
***OARs***					
Lung					
V12.5 (%)	5.83 ± 1.48	5.30 ± 0.85	5.50 ± 0.99	0.420	0.690
V20 (%)	3.05 ± 0.92	2.58 ± 0.71	2.80 ± 0.57	0.820	0.520
Heart					
D_max_ (Gy)	11.15 ± 6.58	9.84 ± 5.89	9.91 ± 6.42	0.058	0.994
Trachea					
D_max_ (Gy)	0.45 ± 0.04	0.55 ± 0.07	0.58 ± 0.06	0.681	0.791
Esophagus					
D_max_ (Gy)	9.14 ± 3.42	8.43 ± 2.14	7.01 ± 2.61	0.457	0.647
Spinal Cord					
D_max_ (Gy)	9.57 ± 2.61	8.89 ± 0.01	6.70 ± 2.45	0.926	0.431
***Treatment efficiency parameters***					
Generate ITV time (min)	30.28 ± 3.74	1.45 ± 0.31	24.13 ± 4.93	756	0.000^*^
MU	1,023.31 ± 83.61	1,059.14 ± 92.38	1,042.36 ± 97.25	0.837	0.713

* *A significant difference existed (p < 0.05)*.

*SD, standard deviation*.

**Figure 4:**
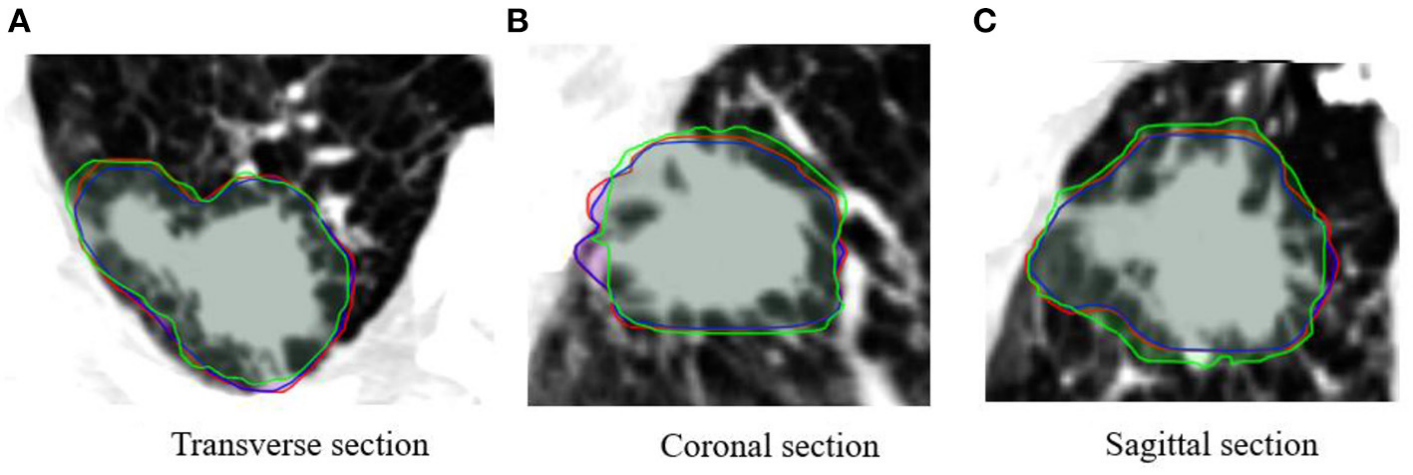
Comparison of contours between planning target volume on four-dimensional CT (PTV_4DCT_) (red), PTV_predict_ (blue), and PTV_FBCT_ (green) in different views of patient images (projected PTV_predict_ and PTV_FBCT_ on 4DCT images).

**Figure 5:**
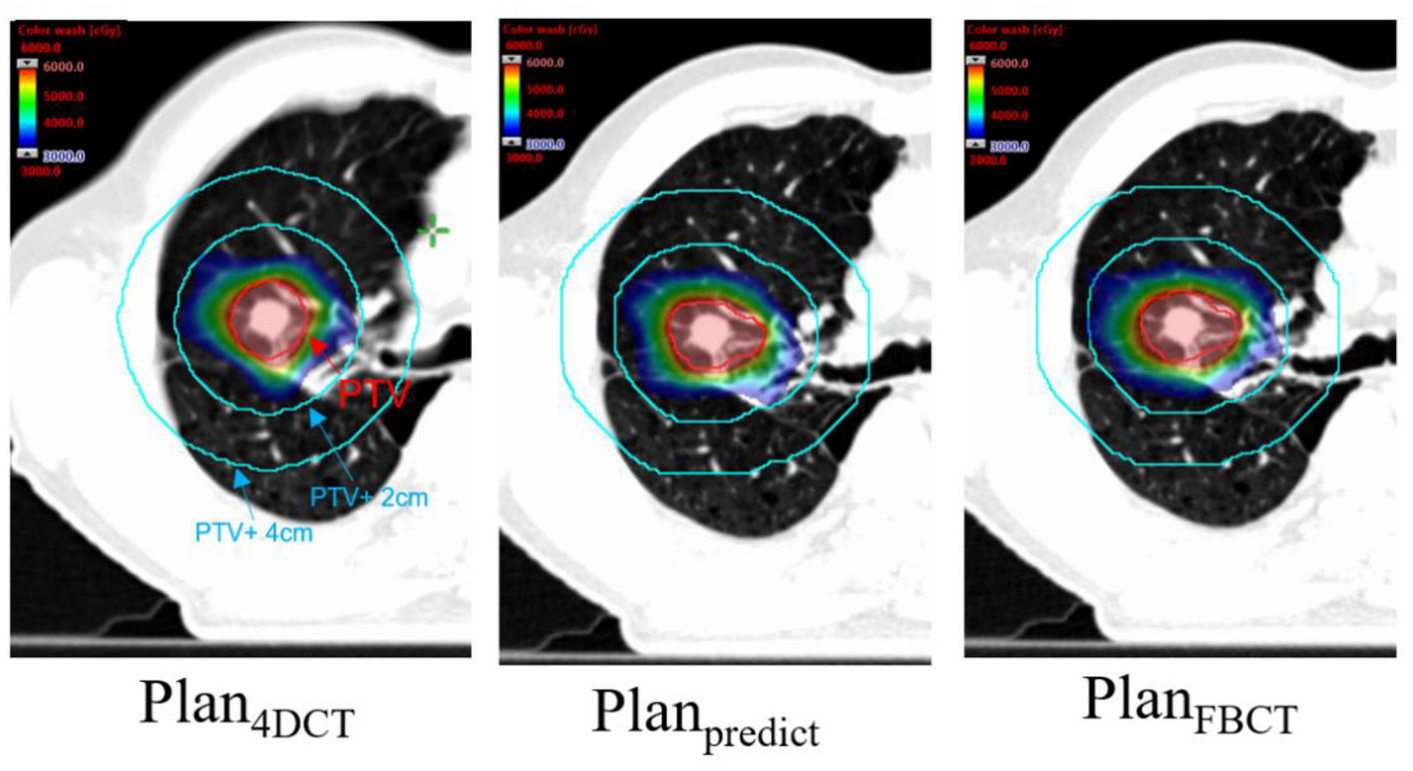
Dose color wash map of each plan for PTV (the area of the red line is PTV, and the red and blue color wash maps represent the 100 and 50% prescription doses, respectively).

**Figure 6:**
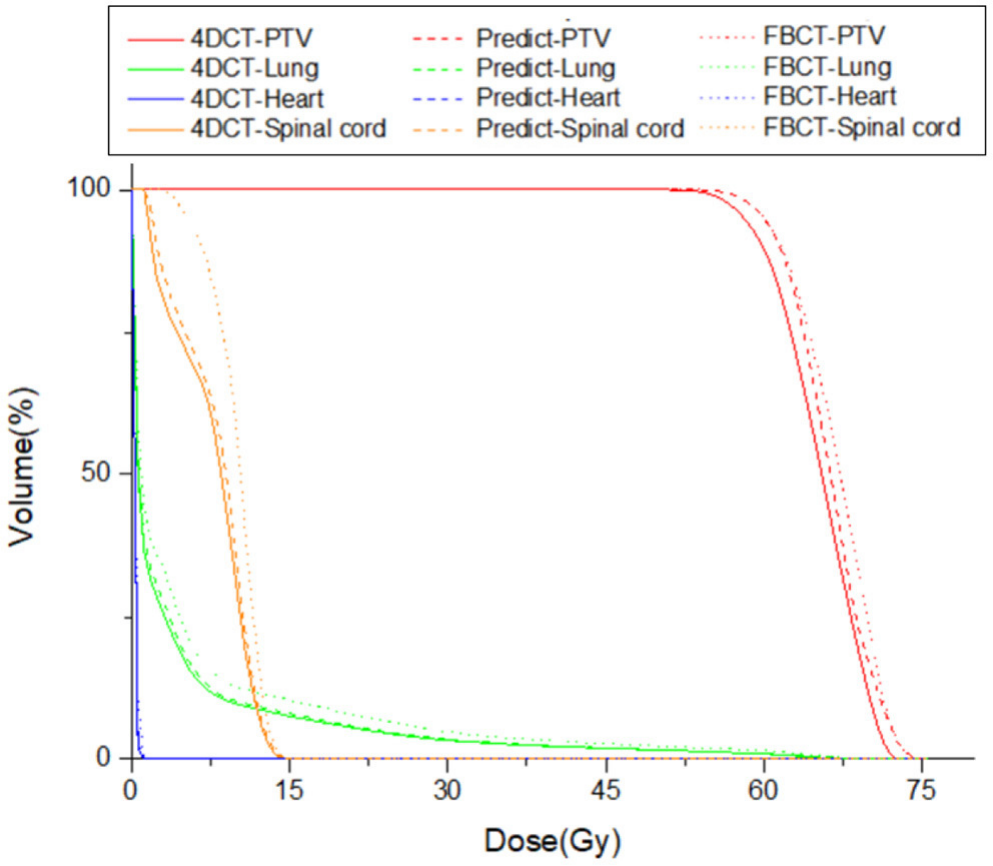
Comparison of the dose-volume histogram (DVH) of one case under the 3 treatment plans: Plan_4DCT_ (solid line), Plan_predict_ (dash line), and Plan_FBCT_ (dot line).

The contouring in different views is also similar for the PTV of each plan (Figure 4). The visual assessment shows that the results are reasonable. Figures 5, 6 show the dose color wash and the dose volume histogram (DVH) of the patients, which were calculated for each plan.

### OARs and Treatment Efficiency Evaluation

**Figure 7:**
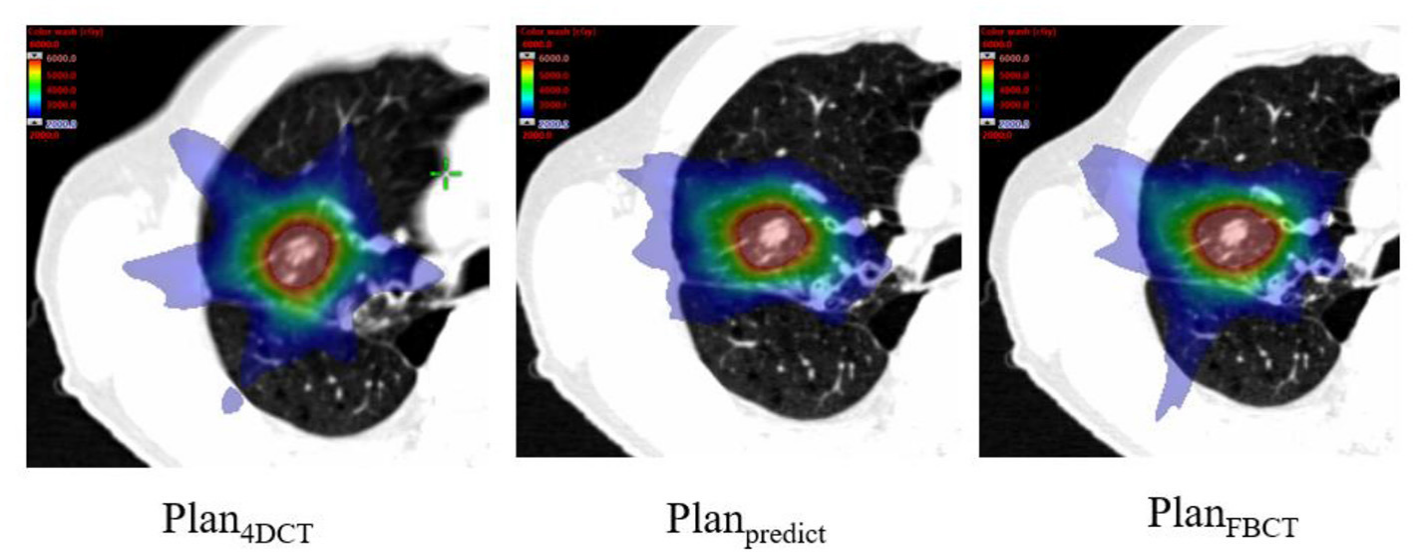
Comparison of color-wash dose distributions of the plans (blue color wash map represents a 20-Gy dose).

The estimated dosimetric parameters for all plans met the criteria specified in the RTOG 0813 protocol. The results show that the V20 of Plan_predict_ was better than that of Plan_4DCT_ (with a reduction of 15.4%) and Plan_FBCT_ (with a reduction of 7.8%), which obviously protected the lung (Figure 7), but no difference existed in the dosimetric parameters. Additionally, there was no statistically significant difference in each plan with the maximum dose (D_max_) for the heart, esophagus, and spinal cord (Table 4).

The average times of generating ITVs were 1.45 ± 0.31 and 30.28 ± 3.74 min for automatic (by model) and manual ITVs, respectively. The model helped reduce it by 95% of the time on average (*p* = 0.000).

## Discussion

### Geometrical Accuracy of the Prediction Model

We used geometric parameters to evaluate the similarity between ITV_4DCT_ and ITV_predict_. The DSC value was 0.83 ± 0.18, showing good agreement between the ITV_predict_ and ITV_4DCT_ contour. DSC > 0.7 is considered to be in good agreement with the gold standard (28, 29). This result can become the basis for follow-up research as it confirmed the feasibility of CBCT images to predict ITV. The accuracy of the ITV contours will directly affect the optimization and calculation of the DVH plan (30). The results show that the ITV_predict_ volume is 10% smaller than the ITV_4DCT_ volume. Dou et al. found that 4DCT images should be used with caution for patients with highly irregular breathing. The simulation indicates that low-pitch helical 4DCT processes potentially yield large tumor motion measurement errors and overestimate tumor motion (31). However, there was no significant difference between ITV_4DCT_ and ITV_predict_. This result of ITV_predict_ volume reduction was acceptable.

### 4DCT Limitation and CBCT Potential Application

Four dimensional CT (4DCT) was acquired during the patient positioning stage and could not reflect the deviation of the tumor's respiratory movement during treatment (32, 33). Rabinowitz et al. showed that during the patient positioning stage and the treatment stage, the tumor deviation caused by respiratory motion was an average of 5.1 mm. For thoracic tumors, the tumor deviation caused by respiratory motion could reach 5.8 mm (34). Yang et al. showed that 4DCT could only collect signals of a limited number of respiratory phases, but the patient's breathing may change at any time and cannot accurately reflect the patient's tumor movement during treatment (35). The study showed that CBCT and maximum intensity projection (MIP) images are equivalent in determining the location of ITV (36). Li et al. showed that 4DCT and CBCT images can indicate variations and inter-fractional setup displacement (37). In our study, we used the first CBCT image at the time of treatment to show the respiratory motion range of a tumor during treatment.

### PTV Evaluation

Compared with conventional treatment, SBRT has higher requirements for the PTV dose gradient and limited dose limitation of organ at risks (OARs). The RTOG0813 protocol (26) provides guidance on the acceptable values of R_100%_, R_50%_, and D_2cm_ based on the PTV volume. The values of R_100%_ and R_50%_ in Plan_4DCT_ and Plan_predict_ were 1.05 ± 0.11, 1.08 ± 0.05, 3.48 ± 0.82, and 5.25 ± 2.01 respectively, which were comparable but slightly different. The result of R_100%_ indicated that Plan_predict_ and Plan_4DCT_ have a similar dose coverage for PTV. The R_50%_ value of Plan_predict_ increased by nearly 30% compared with that of Plan_4DCT_, which shows that Plan_4DCT_ has a stronger ability to constrain PTV. All plans meet the RTOG0813 protocol and can be used in clinical practice.

In our study, we also researched the dosimetry of GTV on FBCT images to generate Plan_FBCT_. The results show that after the RTOG0813 guide on margin from GTV, it can also meet the treatment standards and hence can be used for treatment planning. Tian et al. (38) compared the treatment planning and dose calculation of average intensity projection (AIP) and FBCT for SBRT and concluded that the dosimetric of the two datasets were similar.

### OARs Evaluation

In addition, the RTOG 0813 agreement contains restrictions on each OAR, such as the lung and spinal cord. For SBRT patients, high-energy rays inevitably pass through a part of normal lung tissue, which affects lung function. Jin et al. (39) found that when V20 was >25%, the incidence of radiation pneumonia significantly increased. Our results show that the mean values of V20 in Plan_4DCT_ and Plan_predict_ were 3.05 ± 0.92% and 2.58 ± 0.71%, respectively, indicating that Plan_predict_ reduces the volume of radiation received by normal lung tissue, thereby reducing the incidence of radiation pneumonitis. The thoracic spinal cord is more likely to be injured in patients with lung SBRT. Radiation myelitis may cause serious consequences, such as paraplegia and respiratory paralysis. The values of spinal cord D_max_ in the Plan_4DCT_ and the Plan_predict_ are 9.57 ± 2.61 Gy and 8.89 ± 0.01 Gy. This shows that Plan_predict_ reduces the maximum dose received in the spinal cord, which can thus reduce the incidence of radiation myelitis.

### Treatment Efficiency Evaluation

Currently, the model predicts the ITV volume of large tumors more accurately. For patients with lung SBRT, this model can generate ITV on CBCT images in, on average, 1.45 ± 0.31 min. In our research, the generated ITV time of Plan_predict_ was significantly reduced by nearly 95% compared with that of Plan_4DCT_. Hence, Using the model to input CBCT images can greatly shorten the time to collect patient images and significantly increase the efficiency of tumor delineation for physicians.

This study has some limitations. Firstly, the number of patient samples included was small. Therefore, our patient data did not represent the whole spectrum. Tumors of different sizes and different locations should be included in the future. This could increase patient data for a more uniform tumor volume distribution to ensure accuracy of results in the future.

## Conclusion

Geometric results show that self-generated ITV_predict_ has a good correlation with ITV_4DCT_, although the ITV_predict_ volume is 10% smaller than the ITV_4DCT_ volume. This work confirmed the feasibility of the clinical application of ITV_predict_ to make a treatment plan. There were no significant dosimetry differences between Plan_4DCT_ and Plan_predict_. Thus, our model has potential application in institutions with or without 4DCT scanning technology or when patient breathing is irregular.

## Data Availability Statement

The raw data supporting the conclusions of this article will be made available by the authors, without undue reservation.

## Ethics Statement

Written informed consent was obtained from the individual(s) for the publication of any potentially identifiable images or data included in this article.

## Author Contributions

SZ and BL: conception and design. XZ and YL: acquisition of data. WG, LZ, and FM: analysis of data. SZ, BL, and JQ: writing, review and/or revision of the manuscript. All authors reviewed, read, and approved the final manuscript and authors contributed to the article, and approved the submitted version.

## Funding

This work was partially supported by the National Natural Science Foundation of China (Grant No.11505029 and No.81472794), Shanghai Municipal Commission of Health (Grant Nos. 2018BR23 and 20184Y0099), and Shanghai Municipal Science and Technology Commission (Grant No. 18441904400).

## Conflict of Interest

The authors declare that the research was conducted in the absence of any commercial or financial relationships that could be construed as a potential conflict of interest.

## Publisher's Note

All claims expressed in this article are solely those of the authors and do not necessarily represent those of their affiliated organizations, or those of the publisher, the editors and the reviewers. Any product that may be evaluated in this article, or claim that may be made by its manufacturer, is not guaranteed or endorsed by the publisher.
